# Crystal structures of two different multi-component crystals consisting of 1-(3,4-di­meth­oxy­benz­yl)-6,7-di­meth­oxy­iso­quinoline and fumaric acid

**DOI:** 10.1107/S2056989024009794

**Published:** 2024-10-11

**Authors:** Hiroki Shibata, Aya Sakon, Noriyuki Takata, Hiroshi Takiyama

**Affiliations:** a Pharmaceutical Technology Division, Analytical Development Department, Chugai Pharmaceutical Co. Ltd., 5-5-1 Ukima, Kita-ku, Tokyo 115-8543, Japan; b Department of Applied Physics and Chemical Engineering Tokyo University of Agriculture and Technology 2-24-16 Naka-cho Koganei Tokyo 184-8588 Japan; c Pharmaceutical Technology Division, Formulation Development Department, Chugai Pharmaceutical Co. Ltd., 216 Totsuka-cho, Totsuka-ku, Yokohama, Kanagawa 244-8602, Japan; d Manufacturing Technology Division, Quality Development Department, Chugai Pharma Manufacturing Co. Ltd., 2500 Takayanagi, Fujieda, Shizuoka 426-0041, Japan; University of Hyogo, Japan

**Keywords:** crystal structure, fumaric acid, papaverine, multi-component crystal

## Abstract

Two different multi-component crystals consisting of papaverine [1-(3,4-di­meth­oxy­benz­yl)-6,7-di­meth­oxy­iso­quinoline, C_20_H_21_NO_4_] and fumaric acid [C_4_H_4_O_4_] were obtained. Single-crystal X-ray structure analysis revealed that one, C_20_H_21_NO_4_·1.5C_4_H_4_O_4_ (I), is a salt co-crystal composed of salt-forming and non-salt-forming mol­ecules, and the other, C_20_H_21_NO_4_·0.5C_4_H_4_O_4_ (II), is a salt–co-crystal inter­mediate (*i.e.*, in an inter­mediate state between a salt and a co-crystal). In this study, one state (crystal structure at 100 K) within the salt–co-crystal continuum is defined as the ‘inter­mediate’.

## Abstract 2

Two different multi-component crystals consisting of papaverine [1-(3,4-di­meth­oxy­benz­yl)-6,7-di­meth­oxy­iso­quinoline, C_20_H_21_NO_4_] and fumaric acid [C_4_H_4_O_4_] were obtained.

## 1. Chemical context

Papaverine (1-[3,4-di­meth­oxy­benz­yl]-6,7-di­meth­oxy­iso­quino­line) is an iso­quinoline alkaloid compound extracted from the mature seed capsules of poppies (Kang *et al.*, 2018 ▸). It is an anti­spasmodic and vasodilator, used primarily in the treatment of smooth muscle spasms and for vasodilation and improvement of symptoms in acute arterial embolism, acute pulmonary embolism, peripheral circulatory disturbance, and coronary circulatory disturbance. The active pharmaceutical ingredient papaverine has been developed as a hydro­chloride salt whose crystal structure has been determined (Reynolds *et al.*, 1974 ▸). In the pharmaceutical industry, studies on salt crystallization and co-crystallization are conducted for purposes such as improving the solid-state stability of the active pharmaceutical ingredient or improvement of its dissolution properties. Fumaric acid is a di­carb­oxy­lic acid and a *cis*–*trans* isomer of maleic acid and is used in the pharmaceutical industry as a counter-ion in salts and as a conformer of co-crystals. For example, among 1372 new drugs approved by the US Food and Drug Administration between 1939 and 2020, fumaric acid was used as a counter-ion in the salts of ten drugs (Bharate *et al.*, 2021 ▸). The recently developed COVID-19 anti­viral drug substance Ensitrelvir is crystallized as a co-crystal with fumaric acid (Kawajiri *et al.*, 2023 ▸).

In this work, we synthesized two multicomponent crystals — a salt co-crystal (I) and a salt–co-crystal inter­mediate (II) — consisting of papaverine **1** and fumaric acid **2**, and we determined their crystal structures. In this study, one state (crystal structure at 100 K) within the salt–co-crystal continuum is defined as the ‘inter­mediate’.
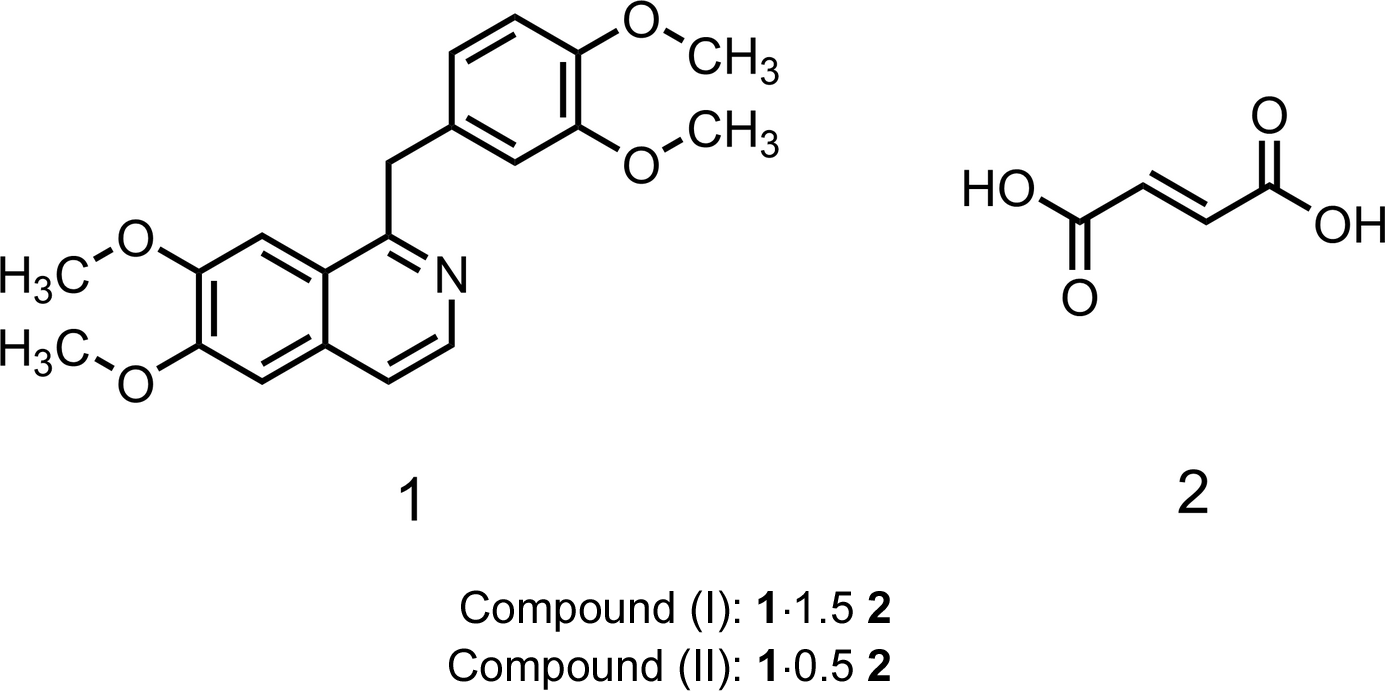


## 2. Structural commentary

**Figure 1:**
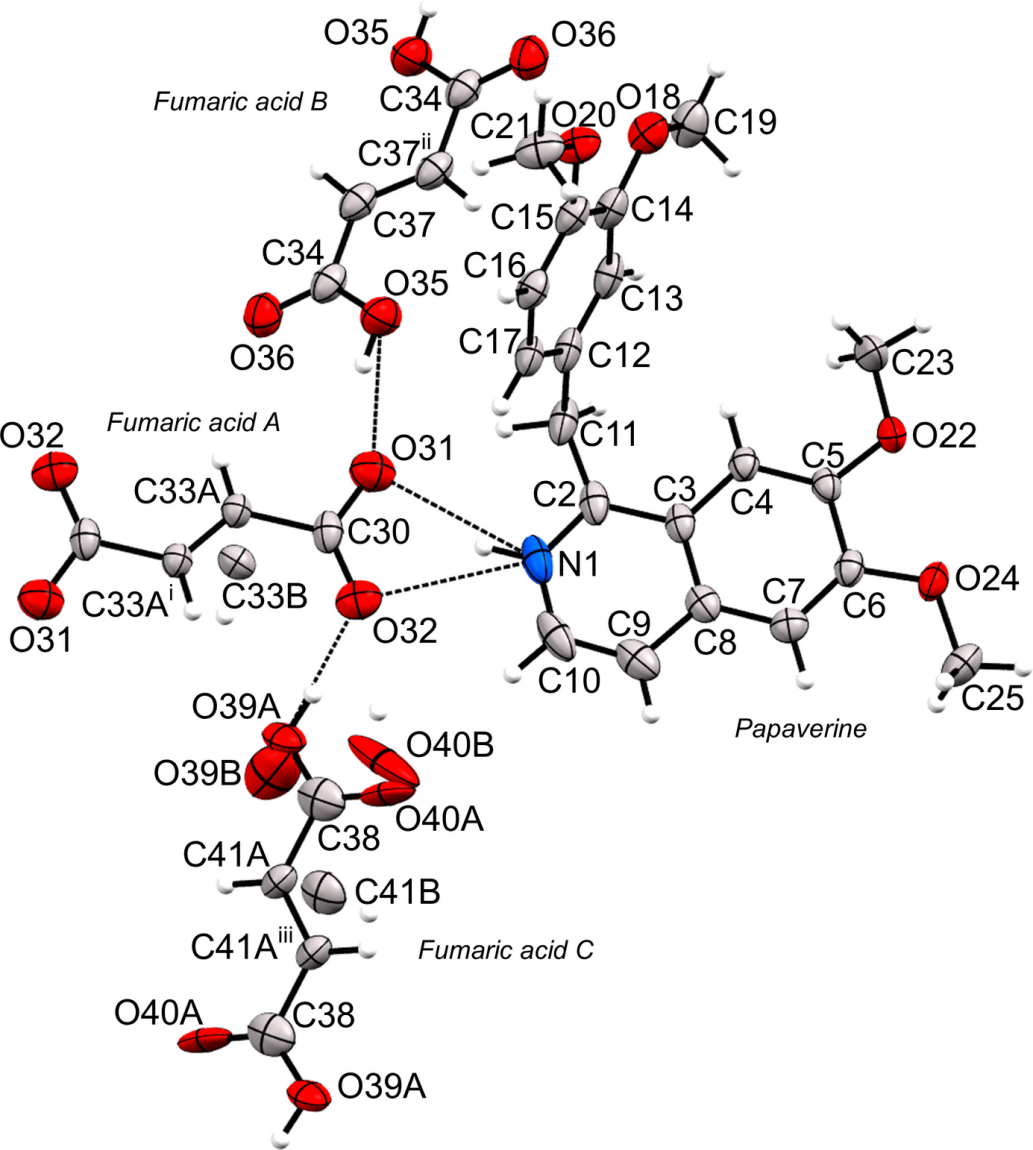
The mol­ecular structure of (I). Hydrogen bonds are shown as dashed lines and displacement ellipsoids are drawn at the 50% probability level. [Symmetry codes: (i) −*x* + 2, −*y* + 2, −*z* + 1; (ii) −*x* + 1, −*y* + 2, −*z*; (iii) −*x* + 2, −*y* + 2, −*z* + 2.]

The crystal structure of (I) is shown in Fig. 1 ▸. It crystallized with a 1:1.5 papaverine:fumaric acid stoichiometric ratio in the space group *P*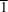 with *Z* = 2, with one full mol­ecule of papaverine and three half mol­ecules of fumaric acid (fumaric acids *A*, *B*, and *C*) in the asymmetric unit. The three fumaric acid mol­ecules were positioned on a center of symmetry, with mol­ecules *A* and *C* being disordered over two positions (O39/O40/C41 and C33).

Since the C30—O31 and C30—O32 distances of fumaric acid mol­ecule *A* are 1.248 (2) Å and 1.246 (3) Å, respectively, the carb­oxy group of mol­ecule *A* is dissociated (Childs *et al.*, 2007 ▸; Chen *et al.*, 2012 ▸). In addition, N1 of the papaverine mol­ecule is protonated and is engaged in N—H⋯O hydrogen bonding (Table 1 ▸). Therefore, it was determined that fumaric acid mol­ecule *A* and the papaverine mol­ecule form a salt. The fumaric acid mol­ecules *B* and *C* are hydrogen-bonded to fumaric acid mol­ecule *A*. The C34—O35 and C34—O36 distances in fumaric acid mol­ecule *B* are 1.324 (3) Å and 1.211 (2) Å, respectively, thus the carb­oxy group of mol­ecule *B* is not dissociated. The C38—O39*A* and C38—O40*A* distances in fumaric acid mol­ecule *C* are 1.280 (5) Å and 1.231 (6) Å, respectively, thus the carb­oxy group of mol­ecule *C* is not dissociated (Childs *et al.*, 2007 ▸; Chen *et al.*, 2012 ▸). Therefore, this multicomponent crystal includes both salt-forming and non–salt-forming mol­ecules and was thus concluded to be a salt co-crystal, (I).

**Figure 2:**
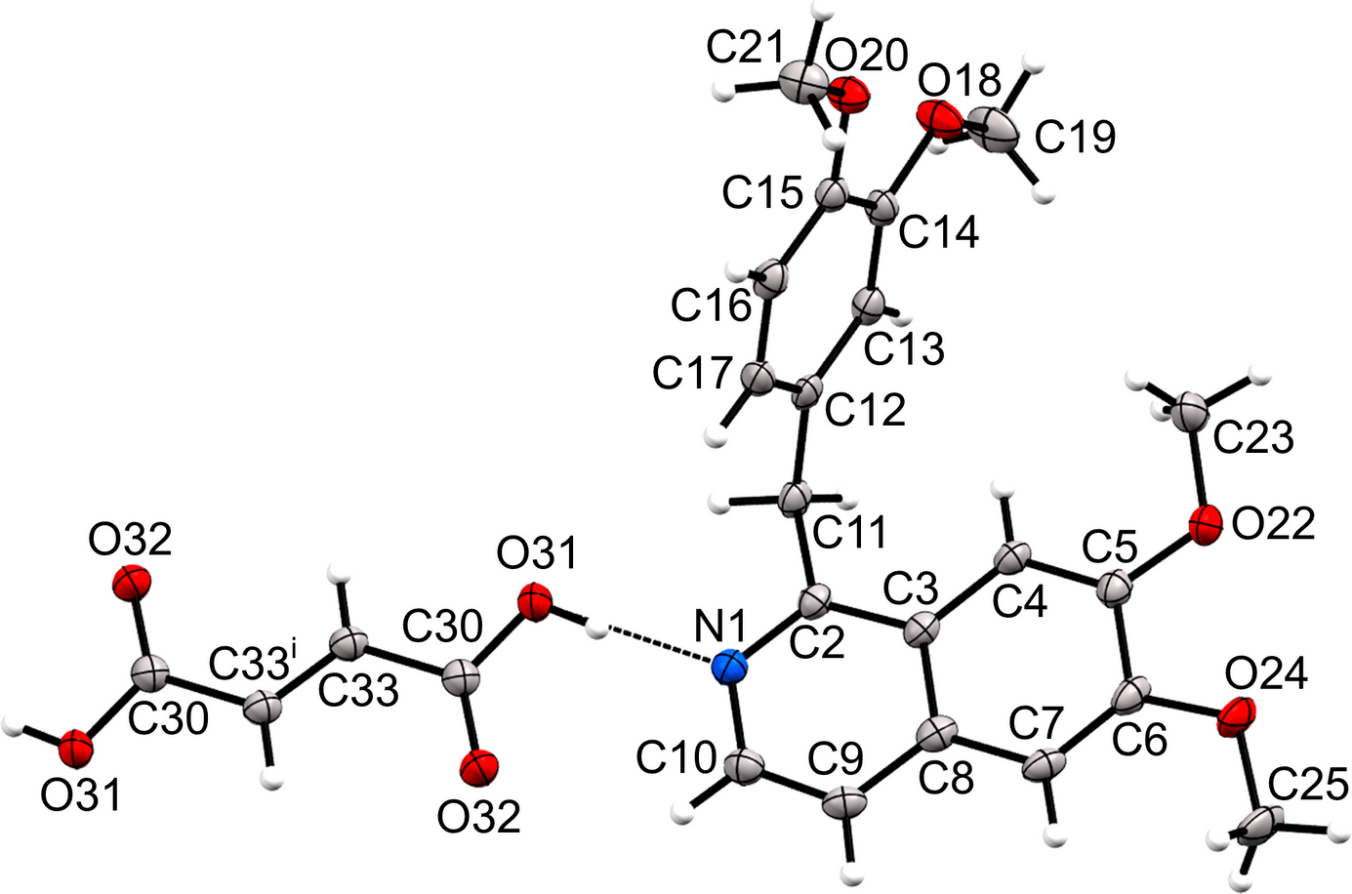
The mol­ecular structure of (II). The hydrogen bond is shown as a dashed line and displacement ellipsoids are drawn at the 50% probability level. [Symmetry code: (i) −*x*, −*y* + 1, −*z* + 1.]

The crystal structure of (II) is given in Fig. 2 ▸. It crystallized with a 1:0.5 papaverine:fumaric acid stoichiometric ratio in space group *P*2_1_/*n* with *Z* = 4, with one full mol­ecule of papaverine and half a mol­ecule of fumaric acid in the asymmetric unit. The fumaric acid mol­ecule is positioned on the center of symmetry. The C30–O31 and C30–O32 distances in the fumaric acid mol­ecule are 1.306 (1) and 1.223 (1) Å, respectively, indicating that the carb­oxy­lic acid of the fumaric acid mol­ecule is not dissociated (Childs *et al.*, 2007 ▸; Chen *et al.*, 2012 ▸). Therefore, the fumaric acid and papaverine mol­ecules were determined to form a co-crystal. However, the O—H⋯N hydrogen bond [*D*⋯*A* = 2.5687 (12) Å, Table 2 ▸) is shorter than that in neutral or ionic synthons, which indicates an inter­mediate state between a salt and a co-crystal (Childs *et al.*, 2007 ▸; Thipparaboina *et al.*, 2015 ▸; Stevens *et al.*, 2020 ▸; Tothadi *et al.*, 2021 ▸; Kotte *et al.*, 2023 ▸). It was thus concluded that this multicomponent crystal, (II), is a salt–co-crystal inter­mediate.

## 3. Supra­molecular features

**Figure 3:**
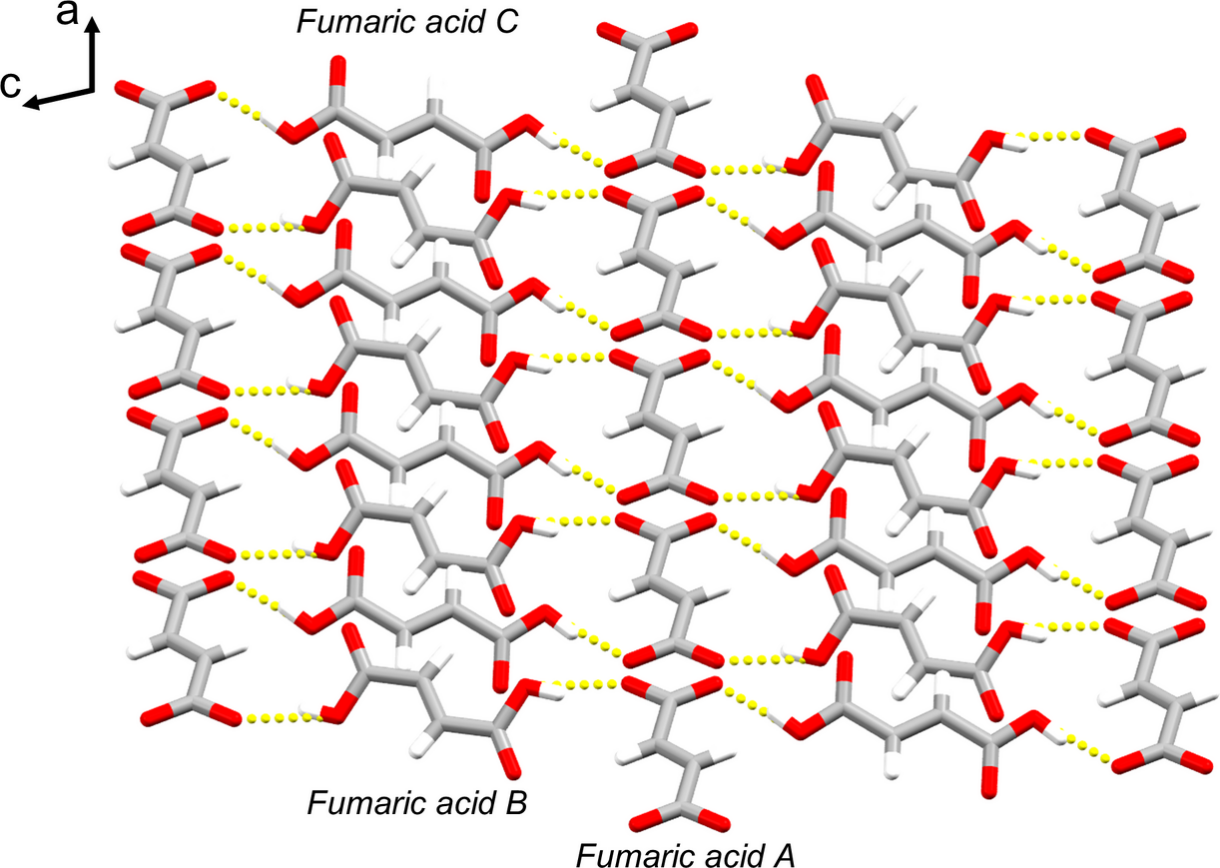
Systematic two-dimensional sheet structure of fumaric acid in (I) viewed along the *ac* plane. Inter­molecular O—H⋯O hydrogen bonds are shown as dashed lines. One of the two disorder components has been omitted for clarity.

**Figure 4:**
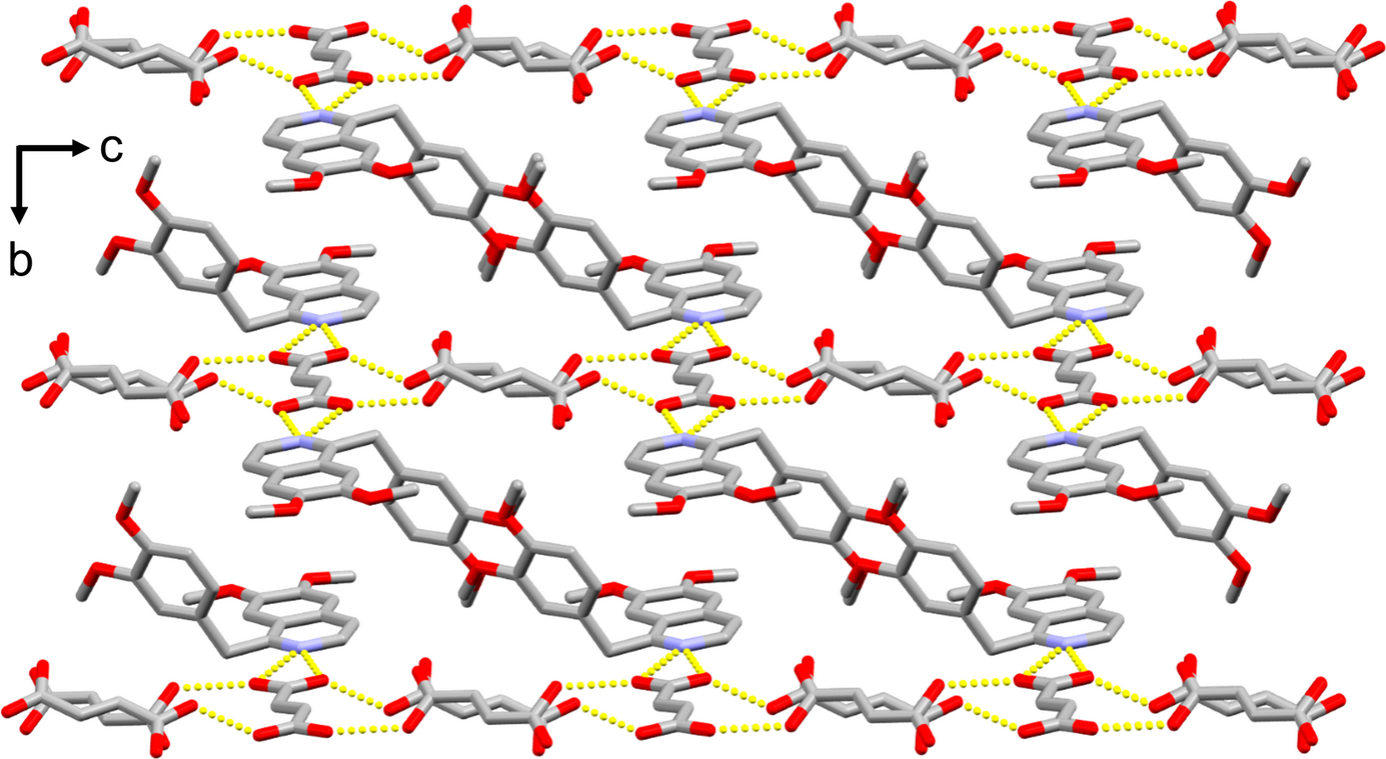
The layered structure of (I) viewed along the *a* axis. Inter­molecular O—H⋯O and N—H⋯O hydrogen bonds are shown as dashed lines. All hydrogen atoms and one of the two disordered components of the fumaric acid mol­ecules have been omitted for clarity.

The combination of the same two components – papaverine and fumaric acid – led to two different multicomponent crystals each with a different stoichiometric ratio and packing. The fumaric acid mol­ecules in (I) form a systematic two-dimensional sheet structure parallel to the *ac* plane with hydrogen bonds linking fumaric acid mol­ecules *A*, *B*, and *C* (Fig. 3 ▸). The space between the fumaric acid sheets is filled with a two-dimensional layer of papaverine mol­ecules hydrogen-bonded to fumaric acid mol­ecules *A*, resulting in (I) having a layered structure (Fig. 4 ▸).

**Figure 5:**
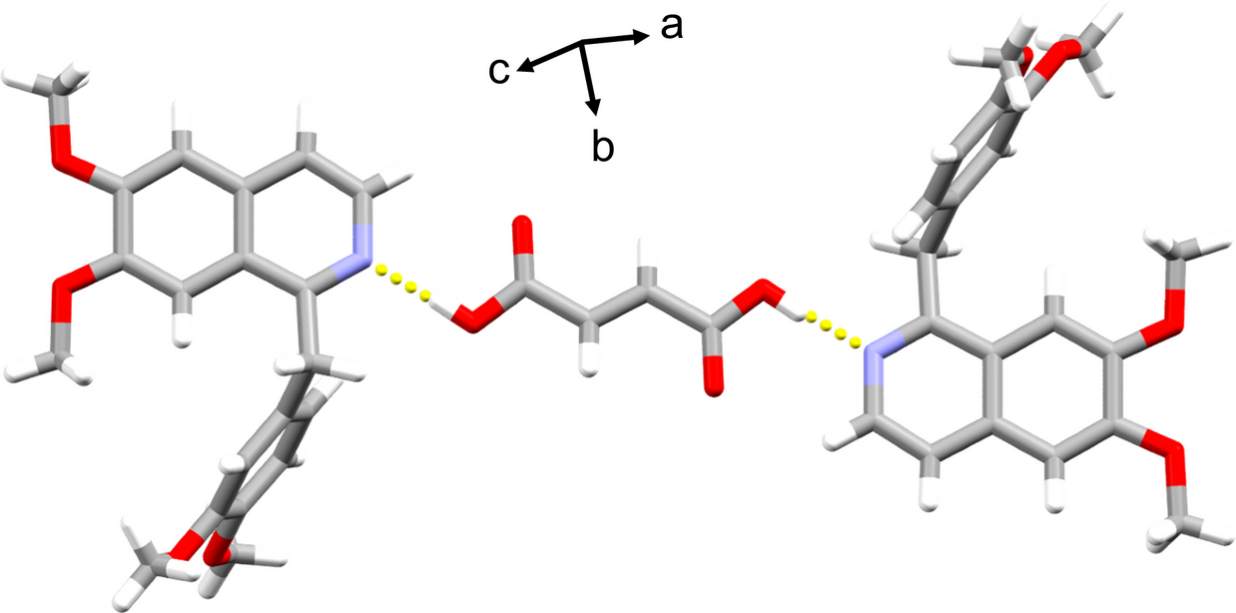
Structural unit in the crystal of (II). Inter­molecular O—H⋯N hydrogen bonds are shown as dashed lines.

**Figure 6:**
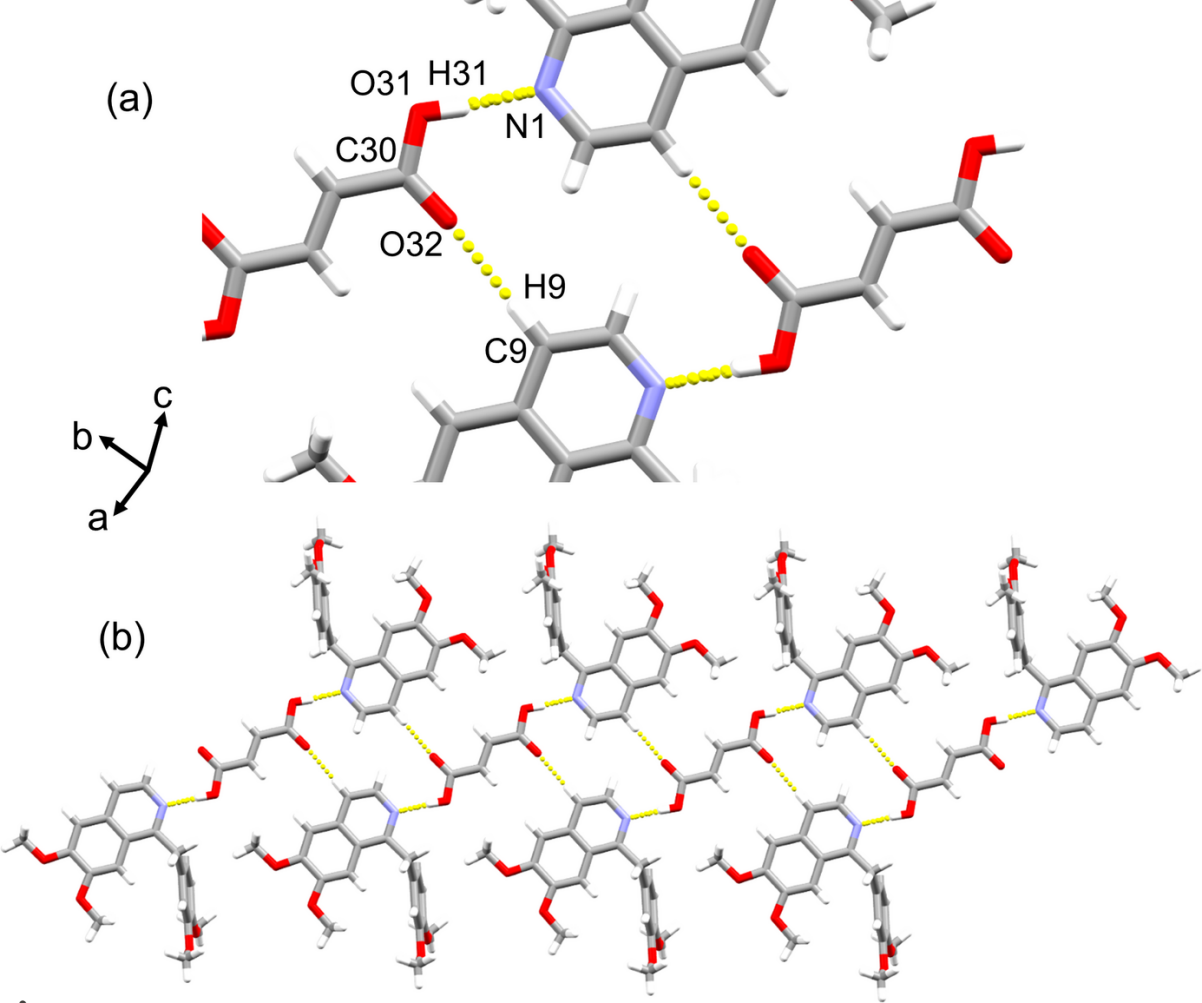
One-dimensional ribbon structure of (II). Inter­molecular O—H⋯N and C—H⋯O hydrogen bonds are shown as dashed lines. (*a*) Enlarged view of hydrogen-bonded ring. (*b*) Overview of the one-dimensional ribbon structure.

**Figure 7:**
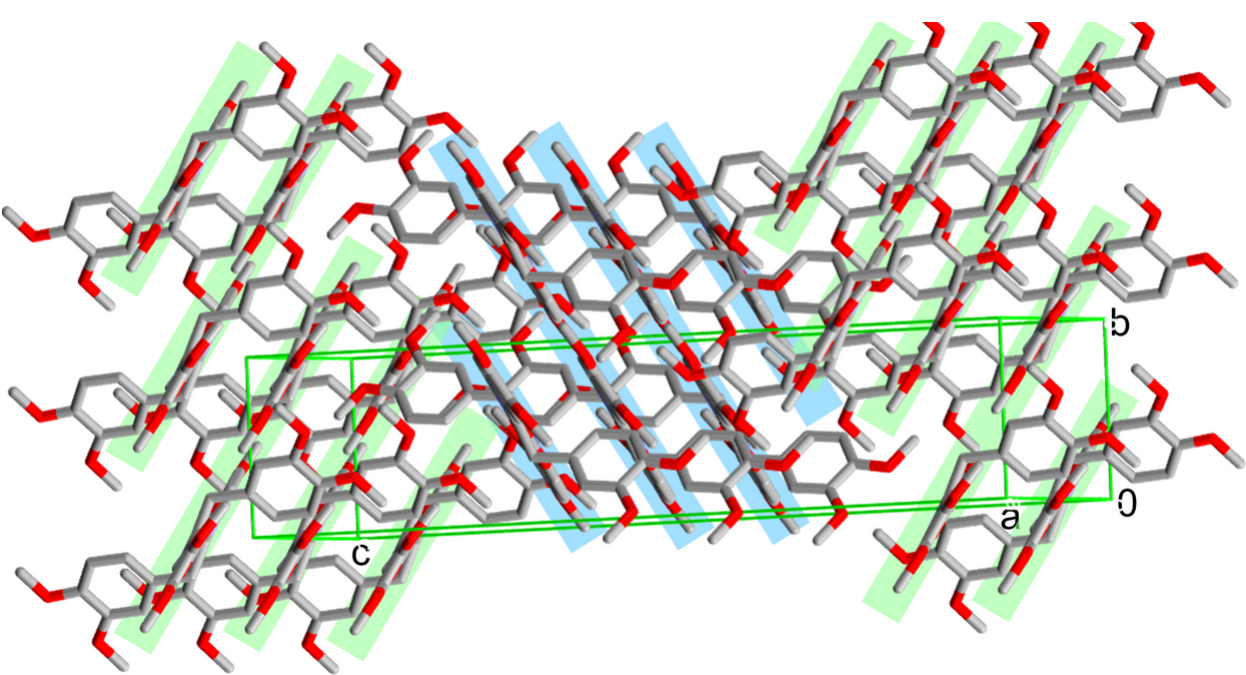
The packing of (II). Each blue and green line represents a one-dimensional ribbon structure. All hydrogen atoms have been removed for clarity.

Compound (II) exhibits a three-mol­ecule unit structure with hydrogen bonds between two papaverine mol­ecules and one fumaric acid mol­ecule (Fig. 5 ▸). The H9⋯O32 and C9⋯O32 distances between two of these three-mol­ecule units are 2.2706 (14) and 3.2191 (14) Å, respectively, with a C9—H9⋯O32 angle of 176.13 (11)° (Fig. 6 ▸*a*, Table 2 ▸); thus, it was concluded that there is a C—H⋯O hydrogen bond (Steiner, 1997 ▸). A ring structure consisting of two O—H⋯N hydrogen bonds and two C—H⋯O hydrogen bonds between two papaverine mol­ecules and two fumaric acid mol­ecules is observed (Fig. 6 ▸*a*). As a result, a one-dimensional ribbon structure is formed by the combination of O—H⋯N and C—H⋯O hydrogen bonds (Fig. 6 ▸*b*). The final crystal structure is formed by the repeated overlapping of these ribbon structures (Fig. 7 ▸).

## 4. Database survey

A survey of the Cambridge Structural Database (WebCSD, v5.44, April 2023; Groom *et al.*, 2016 ▸) for structures with papaverine resulted in four hits. Two crystal structures were free-base, single-component crystals [refcodes MVERIQ (Baggio & Baggio, 1973 ▸) and MVERIQ01 (Marek *et al.*, 1997 ▸)]. The other two crystals were salts: one was a hydro­chloride salt (refcode PAPAVC; Reynolds *et al.*, 1974 ▸) and the other was a hydro­bromide salt (refcode ZZZGYK; Van Hulle *et al.*, 1953 ▸). There were no reports of multi-component crystals of papaverine.

## 5. Synthesis and crystallization

Compound (I) was prepared as follows. About 3 mg (0.009 mmol) of papaverine and 2 mg (0.018 mmol) of fumaric acid were dissolved in 0.025 mL of ethanol. The prepared solution was shaken at room temperature at 100 r.p.m. overnight, and clear light colorless, block-shaped crystals were obtained. Compound (II) was prepared as follows. About 20 mg (0.06 mmol) of papaverine and 10 mg (0.09 mmol) of fumaric acid were dissolved in 0.28 mL of a mixture of acetone and water (6:1) with heating at 368K. The prepared solution was shaken at room temperature at 100 r.p.m. overnight, and clear, light, colorless, block-shaped crystals was obtained.

## 6. Refinement

Crystal data, data collection, and structure refinement details are summarized in Table 3 ▸. The N-bound H atom in (I) was positioned geometrically and refined using a riding model with isotropic displacement parameter *U*_iso_(H) = 1.2*U*_eq_(N). The O-bound H atoms in (I) were located in difference-Fourier maps and refined with O—H = 0.84 Å and with isotropic displacement parameters *U*_iso_(H) = 1.5*U*_eq_(O). The C-bound H atoms in (I) were positioned geometrically (C—H = 0.95, 0.98, and 0.99 Å for *sp*^2^-hybridized, methyl, and methyl­ene hydrogen atoms, respectively) and refined using a riding model, with isotropic displacement parameters *U*_iso_(H) = 1.5*U*_eq_(C) for methyl and *U*_iso_(H) = 1.2*U*_eq_(C) for all other H atoms. The fumaric acid was disordered over two positions (O39/O40/C41 and C33), for which occupancies were refined, converging to 0.598/0.402 and 0.742/0.258, respectively. Restraints by DFIX were applied for C38/O39/O40, O39/O40, O40/H40, O32/H40, and C38/H40. For compound (II), there were no N-bound H atoms or disorders, and the refinement conditions for O-bound H atoms and C-bound H atoms were the same as those for compound (I).

## Supplementary Material

Crystal structure: contains datablock(s) II, I. DOI: 10.1107/S2056989024009794/ox2007sup1.cif

Structure factors: contains datablock(s) II. DOI: 10.1107/S2056989024009794/ox2007IIsup3.hkl

Structure factors: contains datablock(s) I. DOI: 10.1107/S2056989024009794/ox2007Isup2.hkl

Supporting information file. DOI: 10.1107/S2056989024009794/ox2007Isup4.mol

Supporting information file. DOI: 10.1107/S2056989024009794/ox2007IIsup5.mol

Supporting information file. DOI: 10.1107/S2056989024009794/ox2007Isup6.cml

Supporting information file. DOI: 10.1107/S2056989024009794/ox2007IIsup7.cml

CCDC references: 2312143, 2312144

Additional supporting information:  crystallographic information; 3D view; checkCIF report

## Figures and Tables

**Table 1:** Hydrogen-bond geometry (Å, °) for (I)

*D*—H⋯*A*	*D*—H	H⋯*A*	*D*⋯*A*	*D*—H⋯*A*
N1—H1⋯O31	0.88	2.20	3.034 (3)	159
N1—H1⋯O32	0.88	2.18	2.919 (2)	141
O35—H35⋯O31	0.84	1.83	2.617 (2)	156
O39*A*—H39*A*⋯O32	0.84	1.67	2.502 (4)	169

**Table 2:** Hydrogen-bond geometry (Å, °) for (II)

*D*—H⋯*A*	*D*—H	H⋯*A*	*D*⋯*A*	*D*—H⋯*A*
O31—H31⋯N1	0.84 (1)	1.73 (1)	2.5687 (12)	175 (1)
C9—H9⋯O32^i^	0.95 (1)	2.27 (1)	3.2191 (14)	176 (1)

Symmetry code: (i) 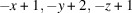.

**Table 3:** Experimental details

	(I)	(II)
Crystal data		
Chemical formula	C_20_H_22_NO_4_·1.5C_4_H_4_O_4_	C_20_H_21_NO_4_·0.5C_4_H_4_O_4_
*M*_r_	513.48	397.43
Crystal system, space group	Triclinic, *P* 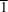	Monoclinic, *P*2_1_/*n*
Temperature (K)	100	100
*a*, *b*, *c* (Å)	9.5290 (2), 10.5445 (3), 12.6509 (4)	9.05718 (12), 6.71363 (11), 32.8419 (4)
α, β, γ (°)	91.606 (2), 104.980 (2), 97.823 (2)	90, 95.9308 (12), 90
*V* (Å^3^)	1213.87 (6)	1986.31 (5)
*Z*	2	4
Radiation type	Cu *K*α	Cu *K*α
μ (mm^−1^)	0.92	0.80
Crystal size (mm)	0.17 × 0.08 × 0.03	0.22 × 0.11 × 0.11

Data collection		
Diffractometer	XtaLAB Synergy, Single source at home/near, HyPix3000	XtaLAB Synergy, Single source at home/near, HyPix3000
Absorption correction	Multi-scan (*CrysAlis PRO*; Rigaku OD, 2022 ▸)	Multi-scan (*CrysAlis PRO*; Rigaku OD, 2022 ▸)
*T*_min_, *T*_max_	0.862, 1.000	0.908, 1.000
No. of measured, independent and observed reflections	10089, 4366, 3457 [*I* > 2σ(*I*)]	8710, 3589, 3325 [*I* ≥ 2u(*I*)]
*R*_int_	0.026	0.018
(sin θ/λ)_max_ (Å^−1^)	0.601	0.601

Refinement		
*R*[*F*^2^ > 2σ(*F*^2^)], *wR*(*F*^2^), *S*	0.047, 0.133, 1.06	0.033, 0.087, 1.04
No. of reflections	4366	3589
No. of parameters	382	268
No. of restraints	282	0
H-atom treatment	H atoms treated by a mixture of independent and constrained refinement	H atoms treated by a mixture of independent and constrained refinement
Δρ_max_, Δρ_min_ (e Å^−3^)	0.38, −0.39	0.27, −0.21

Computer programs: *CrysAlis PRO* (Rigaku OD, 2022 ▸), *SHELXT2018/2* (Sheldrick, 2015*a* ▸), *SHELXL2019/2* (Sheldrick, 2015*b* ▸), *OLEX2.refine* (Bourhis *et al.*, 2015 ▸) and *OLEX2* (Dolomanov *et al.*, 2009 ▸).

## supplementary crystallographic information

### 1-(3,4-Dimethoxybenzyl)-6,7-dimethoxyisoquinoline–fumaric acid (2/1) (II) Crystal data

**Table:**

C_20_H_21_NO_4_·0.5C_4_H_4_O_4_	*F*(000) = 843.079
*M**_r_* = 397.43	*D*_x_ = 1.329 Mg m^−^^3^
Monoclinic, *P*2_1_/*n*	Cu *K*α radiation, λ = 1.54184 Å
*a* = 9.05718 (12) Å	Cell parameters from 6957 reflections
*b* = 6.71363 (11) Å	θ = 2.7–67.9°
*c* = 32.8419 (4) Å	µ = 0.80 mm^−^^1^
β = 95.9308 (12)°	*T* = 100 K
*V* = 1986.31 (5) Å^3^	Block, clear light colourless
*Z* = 4	0.22 × 0.11 × 0.11 mm

### 1-(3,4-Dimethoxybenzyl)-6,7-dimethoxyisoquinoline–fumaric acid (2/1) (II) Data collection

**Table:**

XtaLAB Synergy, Single source at home/near, HyPix3000 diffractometer	3589 independent reflections
Radiation source: micro-focus sealed X-ray tube, PhotonJet (Cu) X-ray Source	3325 reflections with *I*≥ 2u(*I*)
Mirror monochromator	*R*_int_ = 0.018
Detector resolution: 10.0000 pixels mm^-1^	θ_max_ = 68.0°, θ_min_ = 2.7°
ω scans	*h* = −10→5
Absorption correction: multi-scan (CrysAlisPro; Rigaku OD, 2022)	*k* = −8→8
*T*_min_ = 0.908, *T*_max_ = 1.000	*l* = −39→39
8710 measured reflections	

### 1-(3,4-Dimethoxybenzyl)-6,7-dimethoxyisoquinoline–fumaric acid (2/1) (II) Refinement

**Table:**

Refinement on *F*^2^	37 constraints
Least-squares matrix: full	H atoms treated by a mixture of independent and constrained refinement
*R*[*F*^2^ > 2σ(*F*^2^)] = 0.033	*w* = 1/[σ^2^(*F*_o_^2^) + (0.0429*P*)^2^ + 0.6045*P*] where *P* = (*F*_o_^2^ + 2*F*_c_^2^)/3
*wR*(*F*^2^) = 0.087	(Δ/σ)_max_ = −0.0003
*S* = 1.04	Δρ_max_ = 0.27 e Å^−^^3^
3589 reflections	Δρ_min_ = −0.21 e Å^−^^3^
268 parameters	Extinction correction: *SHELXL2019/2* (Sheldrick, 2015b), Fc^*^=kFc[1+0.001xFc^2^λ^3^/sin(2θ)]^-1/4^
0 restraints	Extinction coefficient: 0.0012 (2)

### 1-(3,4-Dimethoxybenzyl)-6,7-dimethoxyisoquinoline–fumaric acid (2/1) (II) Fractional atomic coordinates and isotropic or equivalent isotropic displacement parameters (Å^2^)

**Table:**

	*x*	*y*	*z*	*U*_iso_*/*U*_eq_	
N1	0.51693 (10)	0.60177 (14)	0.44622 (3)	0.0210 (2)	
C2	0.60617 (12)	0.51334 (17)	0.42229 (3)	0.0187 (2)	
C3	0.74048 (11)	0.60359 (17)	0.41307 (3)	0.0182 (2)	
C4	0.83390 (12)	0.51426 (17)	0.38613 (3)	0.0197 (2)	
H4	0.80587 (12)	0.39140 (17)	0.37322 (3)	0.0237 (3)*	
C5	0.96396 (12)	0.60367 (18)	0.37863 (3)	0.0218 (2)	
C6	1.00943 (13)	0.78635 (18)	0.39912 (3)	0.0238 (3)	
C7	0.92008 (13)	0.87524 (17)	0.42489 (3)	0.0229 (3)	
H7	0.94998 (13)	0.99708 (17)	0.43796 (3)	0.0274 (3)*	
C8	0.78282 (12)	0.78679 (17)	0.43229 (3)	0.0200 (2)	
C9	0.68699 (13)	0.87280 (17)	0.45879 (3)	0.0227 (2)	
H9	0.71313 (13)	0.99387 (17)	0.47268 (3)	0.0272 (3)*	
C10	0.55693 (13)	0.77991 (18)	0.46414 (3)	0.0233 (3)	
H10	0.49118 (13)	0.84156 (18)	0.48104 (3)	0.0279 (3)*	
C11	0.56339 (12)	0.30741 (17)	0.40725 (3)	0.0206 (2)	
H11a	0.47821 (12)	0.26165 (17)	0.42142 (3)	0.0247 (3)*	
H11b	0.64743 (12)	0.21623 (17)	0.41512 (3)	0.0247 (3)*	
C12	0.52185 (11)	0.29044 (16)	0.36140 (3)	0.0192 (2)	
C13	0.58166 (12)	0.13608 (17)	0.33954 (3)	0.0205 (2)	
H13	0.64718 (12)	0.04275 (17)	0.35364 (3)	0.0246 (3)*	
C14	0.54649 (12)	0.11766 (17)	0.29756 (3)	0.0208 (2)	
C15	0.44833 (11)	0.25584 (17)	0.27672 (3)	0.0200 (2)	
C16	0.38820 (12)	0.40628 (17)	0.29850 (4)	0.0223 (2)	
H16	0.32136 (12)	0.49876 (17)	0.28463 (4)	0.0268 (3)*	
C17	0.42478 (12)	0.42374 (17)	0.34077 (4)	0.0219 (2)	
H17	0.38266 (12)	0.52785 (17)	0.35539 (4)	0.0262 (3)*	
O18	0.60006 (10)	−0.02676 (13)	0.27362 (3)	0.0286 (2)	
C19	0.70127 (16)	−0.1679 (2)	0.29353 (4)	0.0363 (3)	
H19a	0.7335 (9)	−0.2609 (10)	0.27322 (6)	0.0545 (5)*	
H19b	0.7878 (6)	−0.0977 (3)	0.3070 (3)	0.0545 (5)*	
H19c	0.6519 (4)	−0.2418 (11)	0.3140 (2)	0.0545 (5)*	
O20	0.41893 (9)	0.22626 (12)	0.23544 (2)	0.0241 (2)	
C21	0.32565 (14)	0.37026 (19)	0.21353 (4)	0.0291 (3)	
H21a	0.3150 (9)	0.3372 (8)	0.18431 (5)	0.0436 (4)*	
H21b	0.2278 (4)	0.3696 (10)	0.2238 (2)	0.0436 (4)*	
H21c	0.3702 (6)	0.5028 (3)	0.2175 (2)	0.0436 (4)*	
O22	1.05851 (9)	0.53419 (14)	0.35250 (3)	0.0282 (2)	
C23	1.00672 (13)	0.37103 (19)	0.32693 (4)	0.0289 (3)	
H23a	1.0801 (5)	0.3403 (9)	0.3079 (2)	0.0433 (4)*	
H23b	0.9924 (10)	0.2541 (4)	0.34395 (4)	0.0433 (4)*	
H23c	0.9122 (5)	0.4069 (5)	0.3114 (2)	0.0433 (4)*	
O24	1.14269 (9)	0.85526 (14)	0.39014 (3)	0.0324 (2)	
C25	1.20004 (16)	1.0296 (2)	0.41170 (4)	0.0373 (3)	
H25a	1.2970 (6)	1.0637 (10)	0.4028 (3)	0.0559 (5)*	
H25b	1.1312 (6)	1.1410 (5)	0.4058 (3)	0.0559 (5)*	
H25c	1.2110 (12)	1.0031 (6)	0.44120 (5)	0.0559 (5)*	
C30	0.18953 (12)	0.55150 (17)	0.48065 (3)	0.0206 (2)	
O31	0.26149 (9)	0.45088 (13)	0.45496 (3)	0.0256 (2)	
H31	0.3443 (7)	0.5047 (14)	0.4531 (4)	0.0384 (3)*	
O32	0.23373 (9)	0.70574 (13)	0.49758 (3)	0.0272 (2)	
C33	0.04511 (12)	0.45913 (17)	0.48787 (3)	0.0213 (2)	
H33	0.01702 (12)	0.33727 (17)	0.47454 (3)	0.0256 (3)*	

### 1-(3,4-Dimethoxybenzyl)-6,7-dimethoxyisoquinoline–fumaric acid (2/1) (II) Atomic displacement parameters (Å^2^)

**Table:**

	*U*^11^	*U*^22^	*U*^33^	*U*^12^	*U*^13^	*U*^23^
N1	0.0193 (4)	0.0212 (5)	0.0225 (5)	−0.0011 (4)	0.0023 (4)	−0.0005 (4)
C2	0.0174 (5)	0.0194 (6)	0.0189 (5)	−0.0011 (4)	−0.0002 (4)	0.0019 (4)
C3	0.0178 (5)	0.0175 (5)	0.0188 (5)	−0.0022 (4)	−0.0011 (4)	0.0029 (4)
C4	0.0191 (5)	0.0184 (5)	0.0213 (5)	−0.0041 (4)	0.0002 (4)	0.0003 (4)
C5	0.0196 (5)	0.0252 (6)	0.0207 (5)	−0.0036 (5)	0.0024 (4)	0.0016 (5)
C6	0.0226 (6)	0.0275 (6)	0.0209 (5)	−0.0114 (5)	0.0002 (4)	0.0043 (5)
C7	0.0271 (6)	0.0196 (6)	0.0209 (5)	−0.0084 (5)	−0.0022 (4)	0.0012 (4)
C8	0.0227 (5)	0.0175 (6)	0.0189 (5)	−0.0024 (4)	−0.0025 (4)	0.0035 (4)
C9	0.0280 (6)	0.0178 (6)	0.0214 (5)	−0.0016 (5)	−0.0012 (4)	−0.0014 (4)
C10	0.0251 (6)	0.0211 (6)	0.0236 (6)	0.0010 (5)	0.0024 (4)	−0.0019 (5)
C11	0.0183 (5)	0.0190 (6)	0.0250 (6)	−0.0048 (4)	0.0041 (4)	0.0004 (4)
C12	0.0144 (5)	0.0185 (5)	0.0251 (6)	−0.0061 (4)	0.0034 (4)	−0.0014 (4)
C13	0.0163 (5)	0.0178 (5)	0.0272 (6)	−0.0009 (4)	0.0014 (4)	0.0009 (4)
C14	0.0180 (5)	0.0177 (5)	0.0271 (6)	0.0001 (4)	0.0045 (4)	−0.0026 (4)
C15	0.0172 (5)	0.0194 (5)	0.0234 (5)	−0.0031 (4)	0.0022 (4)	−0.0007 (4)
C16	0.0180 (5)	0.0195 (6)	0.0290 (6)	0.0011 (4)	0.0006 (4)	0.0001 (5)
C17	0.0185 (5)	0.0192 (6)	0.0282 (6)	−0.0003 (4)	0.0040 (4)	−0.0046 (5)
O18	0.0330 (4)	0.0255 (4)	0.0267 (4)	0.0120 (4)	0.0012 (3)	−0.0044 (3)
C19	0.0401 (7)	0.0313 (7)	0.0368 (7)	0.0189 (6)	0.0001 (6)	−0.0051 (6)
O20	0.0265 (4)	0.0225 (4)	0.0228 (4)	0.0041 (3)	0.0006 (3)	−0.0012 (3)
C21	0.0370 (7)	0.0237 (6)	0.0259 (6)	0.0057 (5)	0.0000 (5)	0.0030 (5)
O22	0.0229 (4)	0.0333 (5)	0.0300 (4)	−0.0099 (4)	0.0098 (3)	−0.0059 (4)
C23	0.0282 (6)	0.0286 (7)	0.0314 (6)	−0.0063 (5)	0.0111 (5)	−0.0056 (5)
O24	0.0281 (4)	0.0398 (5)	0.0303 (4)	−0.0218 (4)	0.0071 (3)	−0.0050 (4)
C25	0.0372 (7)	0.0431 (8)	0.0313 (6)	−0.0279 (6)	0.0021 (5)	−0.0028 (6)
C30	0.0217 (5)	0.0200 (6)	0.0200 (5)	−0.0001 (4)	0.0019 (4)	0.0010 (4)
O31	0.0217 (4)	0.0257 (4)	0.0307 (4)	−0.0044 (3)	0.0092 (3)	−0.0064 (3)
O32	0.0252 (4)	0.0246 (5)	0.0326 (4)	−0.0063 (3)	0.0071 (3)	−0.0078 (4)
C33	0.0223 (5)	0.0197 (6)	0.0218 (5)	−0.0019 (4)	0.0014 (4)	−0.0011 (4)

### 1-(3,4-Dimethoxybenzyl)-6,7-dimethoxyisoquinoline–fumaric acid (2/1) (II) Geometric parameters (Å, º)

**Table:**

N1—C2	1.3245 (14)	C15—C16	1.3817 (16)
N1—C10	1.3652 (15)	C15—O20	1.3688 (13)
C2—C3	1.4195 (15)	C16—H16	0.9500
C2—C11	1.5053 (15)	C16—C17	1.3984 (16)
C3—C4	1.4187 (16)	C17—H17	0.9500
C3—C8	1.4172 (16)	O18—C19	1.4288 (15)
C4—H4	0.9500	C19—H19a	0.9800
C4—C5	1.3670 (15)	C19—H19b	0.9800
C5—C6	1.4386 (17)	C19—H19c	0.9800
C5—O22	1.3561 (14)	O20—C21	1.4285 (14)
C6—C7	1.3670 (17)	C21—H21a	0.9800
C6—O24	1.3533 (14)	C21—H21b	0.9800
C7—H7	0.9500	C21—H21c	0.9800
C7—C8	1.4211 (16)	O22—C23	1.4296 (15)
C8—C9	1.4145 (16)	C23—H23a	0.9800
C9—H9	0.9500	C23—H23b	0.9800
C9—C10	1.3602 (17)	C23—H23c	0.9800
C10—H10	0.9500	O24—C25	1.4372 (15)
C11—H11a	0.9900	C25—H25a	0.9800
C11—H11b	0.9900	C25—H25b	0.9800
C11—C12	1.5183 (15)	C25—H25c	0.9800
C12—C13	1.4011 (16)	C30—O31	1.3064 (14)
C12—C17	1.3819 (16)	C30—O32	1.2230 (14)
C13—H13	0.9500	C30—C33	1.4884 (16)
C13—C14	1.3880 (16)	O31—H31	0.8400
C14—C15	1.4120 (16)	C33—C33^i^	1.319 (2)
C14—O18	1.3684 (14)	C33—H33	0.9500
			
C10—N1—C2	119.83 (10)	C16—C15—C14	119.43 (10)
C3—C2—N1	121.62 (10)	O20—C15—C14	115.67 (10)
C11—C2—N1	117.02 (9)	O20—C15—C16	124.90 (10)
C11—C2—C3	121.27 (10)	H16—C16—C15	119.68 (7)
C4—C3—C2	122.15 (10)	C17—C16—C15	120.64 (10)
C8—C3—C2	118.28 (10)	C17—C16—H16	119.68 (7)
C8—C3—C4	119.55 (10)	C16—C17—C12	120.38 (10)
H4—C4—C3	119.75 (6)	H17—C17—C12	119.81 (7)
C5—C4—C3	120.49 (10)	H17—C17—C16	119.81 (7)
C5—C4—H4	119.75 (7)	C19—O18—C14	117.12 (9)
C6—C5—C4	120.02 (10)	H19a—C19—O18	109.5
O22—C5—C4	125.12 (11)	H19b—C19—O18	109.5
O22—C5—C6	114.86 (10)	H19b—C19—H19a	109.5
C7—C6—C5	120.18 (10)	H19c—C19—O18	109.5
O24—C6—C5	114.08 (10)	H19c—C19—H19a	109.5
O24—C6—C7	125.74 (11)	H19c—C19—H19b	109.5
H7—C7—C6	119.75 (7)	C21—O20—C15	116.43 (9)
C8—C7—C6	120.51 (10)	H21a—C21—O20	109.5
C8—C7—H7	119.75 (7)	H21b—C21—O20	109.5
C7—C8—C3	119.20 (10)	H21b—C21—H21a	109.5
C9—C8—C3	118.26 (10)	H21c—C21—O20	109.5
C9—C8—C7	122.54 (10)	H21c—C21—H21a	109.5
H9—C9—C8	120.43 (6)	H21c—C21—H21b	109.5
C10—C9—C8	119.14 (10)	C23—O22—C5	116.42 (9)
C10—C9—H9	120.43 (7)	H23a—C23—O22	109.5
C9—C10—N1	122.73 (11)	H23b—C23—O22	109.5
H10—C10—N1	118.63 (6)	H23b—C23—H23a	109.5
H10—C10—C9	118.63 (7)	H23c—C23—O22	109.5
H11a—C11—C2	108.51 (6)	H23c—C23—H23a	109.5
H11b—C11—C2	108.51 (6)	H23c—C23—H23b	109.5
H11b—C11—H11a	107.5	C25—O24—C6	117.14 (10)
C12—C11—C2	115.05 (9)	H25a—C25—O24	109.5
C12—C11—H11a	108.51 (5)	H25b—C25—O24	109.5
C12—C11—H11b	108.51 (6)	H25b—C25—H25a	109.5
C13—C12—C11	119.63 (10)	H25c—C25—O24	109.5
C17—C12—C11	121.16 (10)	H25c—C25—H25a	109.5
C17—C12—C13	119.20 (10)	H25c—C25—H25b	109.5
H13—C13—C12	119.55 (6)	O32—C30—O31	124.73 (10)
C14—C13—C12	120.90 (10)	C33—C30—O31	113.12 (10)
C14—C13—H13	119.55 (7)	C33—C30—O32	122.15 (10)
C15—C14—C13	119.43 (10)	H31—O31—C30	109.5
O18—C14—C13	125.11 (10)	C33^i^—C33—C30	122.17 (13)
O18—C14—C15	115.46 (10)	H33—C33—C30	118.91 (6)
			
N1—C2—C3—C4	−177.32 (10)	C5—C6—C7—C8	1.10 (13)
N1—C2—C3—C8	4.06 (12)	C5—C6—O24—C25	−175.75 (11)
N1—C2—C11—C12	113.81 (10)	C6—C7—C8—C9	179.76 (11)
N1—C10—C9—C8	2.72 (13)	C7—C8—C9—C10	179.60 (11)
C2—C3—C4—C5	−178.66 (11)	C11—C12—C13—C14	−179.55 (10)
C2—C3—C8—C7	177.22 (10)	C11—C12—C17—C16	179.76 (10)
C2—C3—C8—C9	−1.70 (12)	C12—C13—C14—C15	−0.40 (12)
C2—C11—C12—C13	133.10 (9)	C12—C13—C14—O18	179.50 (10)
C2—C11—C12—C17	−47.48 (11)	C12—C17—C16—C15	0.01 (13)
C3—C4—C5—C6	2.05 (12)	C13—C14—C15—C16	−0.41 (12)
C3—C4—C5—O22	−177.78 (10)	C13—C14—C15—O20	−179.63 (10)
C3—C8—C7—C6	0.89 (12)	C13—C14—O18—C19	−0.69 (14)
C3—C8—C9—C10	−1.52 (12)	C14—C15—C16—C17	0.61 (12)
C4—C5—C6—C7	−2.60 (13)	C14—C15—O20—C21	−177.02 (10)
C4—C5—C6—O24	177.69 (11)	C30—C33—C33^i^—C30^i^	180.00 (14)
C4—C5—O22—C23	9.21 (14)		

Symmetry code: (i) −*x*, −*y*+1, −*z*+1.

### 1-(3,4-Dimethoxybenzyl)-6,7-dimethoxyisoquinoline–fumaric acid (2/1) (II) Hydrogen-bond geometry (Å, º)

**Table:**

*D*—H···*A*	*D*—H	H···*A*	*D*···*A*	*D*—H···*A*
O31—H31···N1	0.84 (1)	1.73 (1)	2.5687 (12)	175 (1)
C9—H9···O32^ii^	0.95 (1)	2.27 (1)	3.2191 (14)	176 (1)

Symmetry code: (ii) −*x*+1, −*y*+2, −*z*+1.

### 1-(3,4-Dimethoxybenzyl)-6,7-dimethoxyisoquinoline–fumaric acid (2/3) (I) Crystal data

**Table:**

C_20_H_22_NO_4_·1.5C_4_H_4_O_4_	*Z* = 2
*M**_r_* = 513.48	*F*(000) = 540
Triclinic, *P*1	*D*_x_ = 1.405 Mg m^−^^3^
*a* = 9.5290 (2) Å	Cu *K*α radiation, λ = 1.54184 Å
*b* = 10.5445 (3) Å	Cell parameters from 6061 reflections
*c* = 12.6509 (4) Å	θ = 3.6–68.1°
α = 91.606 (2)°	µ = 0.92 mm^−^^1^
β = 104.980 (2)°	*T* = 100 K
γ = 97.823 (2)°	Block, clear light colourless
*V* = 1213.87 (6) Å^3^	0.17 × 0.08 × 0.03 mm

### 1-(3,4-Dimethoxybenzyl)-6,7-dimethoxyisoquinoline–fumaric acid (2/3) (I) Data collection

**Table:**

XtaLAB Synergy, Single source at home/near, HyPix3000 diffractometer	4366 independent reflections
Radiation source: micro-focus sealed X-ray tube, PhotonJet (Cu) X-ray Source	3457 reflections with *I* > 2σ(*I*)
Mirror monochromator	*R*_int_ = 0.026
Detector resolution: 10.0000 pixels mm^-1^	θ_max_ = 68.0°, θ_min_ = 3.6°
ω scans	*h* = −11→5
Absorption correction: multi-scan (CrysAlisPro; Rigaku OD, 2022)	*k* = −12→12
*T*_min_ = 0.862, *T*_max_ = 1.000	*l* = −15→15
10089 measured reflections	

### 1-(3,4-Dimethoxybenzyl)-6,7-dimethoxyisoquinoline–fumaric acid (2/3) (I) Refinement

**Table:**

Refinement on *F*^2^	Hydrogen site location: mixed
Least-squares matrix: full	H atoms treated by a mixture of independent and constrained refinement
*R*[*F*^2^ > 2σ(*F*^2^)] = 0.047	*w* = 1/[σ^2^(*F*_o_^2^) + (0.0609*P*)^2^ + 0.410*P*] where *P* = (*F*_o_^2^ + 2*F*_c_^2^)/3
*wR*(*F*^2^) = 0.133	(Δ/σ)_max_ < 0.001
*S* = 1.06	Δρ_max_ = 0.38 e Å^−^^3^
4366 reflections	Δρ_min_ = −0.39 e Å^−^^3^
382 parameters	Extinction correction: *SHELXL2019/2* (Sheldrick, 2015b), Fc^*^=kFc[1+0.001xFc^2^λ^3^/sin(2θ)]^-1/4^
282 restraints	Extinction coefficient: 0.0009 (3)
Primary atom site location: structure-invariant direct methods	

### 1-(3,4-Dimethoxybenzyl)-6,7-dimethoxyisoquinoline–fumaric acid (2/3) (I) Special details

**Table:**

Geometry. All esds (except the esd in the dihedral angle between two l.s. planes) are estimated using the full covariance matrix. The cell esds are taken into account individually in the estimation of esds in distances, angles and torsion angles; correlations between esds in cell parameters are only used when they are defined by crystal symmetry. An approximate (isotropic) treatment of cell esds is used for estimating esds involving l.s. planes.

### 1-(3,4-Dimethoxybenzyl)-6,7-dimethoxyisoquinoline–fumaric acid (2/3) (I) Fractional atomic coordinates and isotropic or equivalent isotropic displacement parameters (Å^2^)

**Table:**

	*x*	*y*	*z*	*U*_iso_*/*U*_eq_	Occ. (<1)
N1	0.46197 (17)	0.81758 (17)	0.51609 (18)	0.0444 (5)	
H1	0.543685	0.850236	0.500466	0.053*	
C2	0.3393 (2)	0.79572 (18)	0.43509 (19)	0.0359 (5)	
C3	0.20717 (19)	0.74256 (17)	0.45885 (17)	0.0303 (4)	
C4	0.07358 (18)	0.71542 (17)	0.37492 (16)	0.0286 (4)	
H4	0.073022	0.730035	0.301067	0.034*	
C5	−0.05396 (18)	0.66853 (17)	0.39956 (16)	0.0289 (4)	
C6	−0.05444 (19)	0.64893 (17)	0.51115 (17)	0.0301 (4)	
C7	0.0738 (2)	0.67300 (17)	0.59334 (17)	0.0326 (4)	
H7	0.072747	0.659166	0.667034	0.039*	
C8	0.2077 (2)	0.71842 (17)	0.56852 (17)	0.0320 (4)	
C9	0.3442 (2)	0.7438 (2)	0.6496 (2)	0.0430 (5)	
H9	0.349081	0.726778	0.723680	0.052*	
C10	0.4681 (2)	0.7924 (2)	0.6211 (2)	0.0492 (6)	
H10	0.559426	0.808756	0.675412	0.059*	
C11	0.3489 (2)	0.83103 (18)	0.3232 (2)	0.0404 (5)	
H11A	0.440287	0.891577	0.330194	0.049*	
H11B	0.264971	0.875706	0.289528	0.049*	
C12	0.3477 (2)	0.71580 (18)	0.24786 (19)	0.0356 (5)	
C13	0.2463 (2)	0.69676 (18)	0.14486 (19)	0.0359 (5)	
H13	0.176960	0.754304	0.123955	0.043*	
C14	0.2456 (2)	0.59499 (19)	0.07283 (18)	0.0352 (4)	
C15	0.3481 (2)	0.50903 (18)	0.10481 (17)	0.0338 (4)	
C16	0.4475 (2)	0.52812 (18)	0.20657 (17)	0.0342 (4)	
H16	0.515980	0.470031	0.228377	0.041*	
C17	0.4487 (2)	0.63203 (18)	0.27815 (18)	0.0343 (4)	
H17	0.518893	0.645061	0.347593	0.041*	
O18	0.15271 (16)	0.56999 (14)	−0.02976 (13)	0.0416 (4)	
C19	0.0454 (2)	0.6537 (2)	−0.0642 (2)	0.0451 (5)	
H19A	−0.018009	0.622919	−0.137013	0.068*	
H19B	0.095157	0.740628	−0.067495	0.068*	
H19C	−0.014298	0.654835	−0.011816	0.068*	
O20	0.33975 (16)	0.41301 (14)	0.02746 (12)	0.0415 (4)	
C21	0.4403 (3)	0.3231 (2)	0.05620 (19)	0.0458 (5)	
H21A	0.429616	0.263692	−0.007301	0.069*	
H21B	0.419471	0.274827	0.116846	0.069*	
H21C	0.540978	0.369063	0.078816	0.069*	
O22	−0.18676 (13)	0.63998 (14)	0.32630 (12)	0.0370 (3)	
C23	−0.1929 (2)	0.6669 (3)	0.21502 (19)	0.0514 (6)	
H23A	−0.295060	0.649292	0.170510	0.077*	
H23B	−0.133712	0.612563	0.186378	0.077*	
H23C	−0.154234	0.757365	0.212015	0.077*	
O24	−0.18929 (14)	0.60799 (13)	0.52392 (12)	0.0375 (3)	
C25	−0.2027 (3)	0.6004 (2)	0.63391 (19)	0.0451 (5)	
H25A	−0.305854	0.573898	0.632334	0.068*	
H25B	−0.167756	0.684678	0.673616	0.068*	
H25C	−0.143486	0.537523	0.670996	0.068*	
C34	0.6558 (2)	1.03159 (19)	0.13490 (18)	0.0369 (5)	
O35	0.60430 (18)	0.93880 (15)	0.18930 (13)	0.0484 (4)	
H35	0.654420	0.946279	0.254974	0.073*	
O36	0.76487 (17)	1.10891 (15)	0.17335 (14)	0.0477 (4)	
C37	0.5660 (2)	1.03429 (18)	0.02071 (18)	0.0368 (5)	
H37	0.605453	1.088822	−0.026367	0.044*	
C38	0.9160 (2)	0.9597 (2)	0.8450 (2)	0.0504 (6)	
O39A	0.9425 (5)	1.0085 (3)	0.7594 (3)	0.0410 (9)	0.598 (9)
H39A	0.883696	0.969476	0.703076	0.062*	0.598 (9)
O39B	0.9637 (9)	1.0502 (9)	0.7980 (9)	0.091 (3)	0.402 (9)
O40A	0.8223 (6)	0.8669 (5)	0.8449 (3)	0.0554 (11)	0.598 (9)
O40B	0.8172 (9)	0.8612 (7)	0.8033 (9)	0.095 (3)	0.402 (9)
H40B	0.804 (10)	0.868 (7)	0.7354 (12)	0.142*	0.402 (9)
C41A	1.0105 (5)	1.0188 (5)	0.9512 (4)	0.0314 (11)	0.598 (9)
H41A	1.088580	1.085283	0.951450	0.038*	0.598 (9)
C41B	0.9522 (9)	0.9608 (9)	0.9704 (8)	0.047 (2)	0.402 (9)
H41B	0.898144	0.898230	1.002913	0.057*	0.402 (9)
C30	0.7944 (2)	0.94153 (17)	0.48749 (18)	0.0334 (4)	
O31	0.69127 (18)	0.92626 (15)	0.40213 (14)	0.0491 (4)	
O32	0.77542 (17)	0.91662 (14)	0.57896 (13)	0.0448 (4)	
C33A	0.9361 (4)	0.9954 (2)	0.4628 (4)	0.0278 (11)	0.742 (11)
H33A	0.933667	1.023721	0.391822	0.033*	0.742 (11)
C33B	0.9632 (11)	0.9786 (7)	0.5352 (10)	0.030 (3)	0.258 (11)
H33B	1.010435	0.971108	0.610235	0.036*	0.258 (11)

### 1-(3,4-Dimethoxybenzyl)-6,7-dimethoxyisoquinoline–fumaric acid (2/3) (I) Atomic displacement parameters (Å^2^)

**Table:**

	*U*^11^	*U*^22^	*U*^33^	*U*^12^	*U*^13^	*U*^23^
N1	0.0222 (8)	0.0367 (9)	0.0710 (14)	0.0039 (6)	0.0085 (8)	−0.0155 (9)
C2	0.0237 (9)	0.0261 (9)	0.0583 (13)	0.0035 (7)	0.0131 (9)	−0.0100 (8)
C3	0.0228 (8)	0.0239 (8)	0.0446 (12)	0.0056 (6)	0.0094 (8)	−0.0053 (8)
C4	0.0246 (8)	0.0280 (9)	0.0347 (10)	0.0044 (7)	0.0105 (8)	−0.0023 (7)
C5	0.0223 (8)	0.0281 (9)	0.0354 (10)	0.0033 (7)	0.0072 (7)	−0.0049 (7)
C6	0.0272 (9)	0.0252 (9)	0.0406 (11)	0.0032 (7)	0.0145 (8)	−0.0021 (8)
C7	0.0372 (10)	0.0276 (9)	0.0349 (11)	0.0093 (7)	0.0107 (8)	0.0002 (8)
C8	0.0286 (9)	0.0263 (9)	0.0402 (11)	0.0097 (7)	0.0052 (8)	−0.0047 (8)
C9	0.0397 (11)	0.0355 (11)	0.0476 (13)	0.0164 (8)	−0.0039 (9)	−0.0101 (9)
C10	0.0257 (10)	0.0407 (12)	0.0709 (17)	0.0117 (8)	−0.0066 (10)	−0.0190 (11)
C11	0.0317 (10)	0.0281 (9)	0.0681 (15)	−0.0007 (7)	0.0282 (10)	−0.0027 (9)
C12	0.0284 (9)	0.0271 (9)	0.0590 (14)	0.0022 (7)	0.0260 (9)	0.0019 (9)
C13	0.0296 (9)	0.0301 (9)	0.0567 (13)	0.0094 (7)	0.0236 (9)	0.0100 (9)
C14	0.0332 (10)	0.0347 (10)	0.0450 (12)	0.0082 (8)	0.0211 (9)	0.0106 (8)
C15	0.0362 (10)	0.0319 (9)	0.0418 (12)	0.0106 (8)	0.0218 (9)	0.0071 (8)
C16	0.0334 (9)	0.0323 (10)	0.0445 (12)	0.0113 (7)	0.0199 (9)	0.0060 (8)
C17	0.0270 (9)	0.0332 (10)	0.0475 (12)	0.0029 (7)	0.0197 (8)	0.0002 (8)
O18	0.0433 (8)	0.0432 (8)	0.0438 (9)	0.0172 (6)	0.0150 (7)	0.0102 (7)
C19	0.0401 (11)	0.0467 (12)	0.0550 (14)	0.0166 (9)	0.0175 (10)	0.0168 (11)
O20	0.0536 (8)	0.0386 (8)	0.0389 (8)	0.0213 (7)	0.0164 (7)	0.0036 (6)
C21	0.0624 (14)	0.0410 (12)	0.0420 (13)	0.0261 (10)	0.0182 (11)	0.0036 (10)
O22	0.0212 (6)	0.0521 (8)	0.0353 (8)	0.0007 (5)	0.0062 (5)	−0.0040 (6)
C23	0.0283 (10)	0.0872 (18)	0.0347 (12)	0.0023 (10)	0.0051 (9)	0.0011 (12)
O24	0.0320 (7)	0.0389 (7)	0.0457 (9)	−0.0017 (5)	0.0213 (6)	−0.0004 (6)
C25	0.0530 (13)	0.0430 (12)	0.0507 (14)	0.0094 (10)	0.0322 (11)	0.0067 (10)
C34	0.0422 (11)	0.0301 (10)	0.0452 (12)	0.0068 (8)	0.0227 (9)	0.0024 (8)
O35	0.0550 (9)	0.0476 (9)	0.0401 (9)	−0.0056 (7)	0.0147 (7)	0.0075 (7)
O36	0.0445 (8)	0.0464 (9)	0.0528 (10)	−0.0001 (7)	0.0169 (7)	0.0068 (7)
C37	0.0462 (11)	0.0294 (9)	0.0420 (12)	0.0073 (8)	0.0237 (9)	0.0034 (8)
C38	0.0472 (13)	0.0539 (14)	0.0506 (15)	0.0289 (11)	0.0036 (11)	−0.0042 (11)
O39A	0.0466 (17)	0.0416 (16)	0.0310 (18)	0.0077 (12)	0.0045 (15)	−0.0136 (12)
O39B	0.069 (4)	0.150 (6)	0.068 (6)	0.054 (5)	0.020 (4)	0.054 (5)
O40A	0.079 (3)	0.075 (3)	0.0154 (18)	0.0210 (18)	0.0145 (19)	0.0043 (17)
O40B	0.086 (4)	0.082 (4)	0.082 (6)	0.042 (3)	−0.048 (4)	−0.058 (4)
C41A	0.034 (2)	0.035 (2)	0.031 (3)	0.0118 (16)	0.0154 (18)	0.0083 (17)
C41B	0.043 (4)	0.050 (4)	0.048 (5)	0.021 (3)	0.002 (3)	0.005 (3)
C30	0.0262 (9)	0.0246 (9)	0.0509 (13)	0.0034 (7)	0.0140 (9)	−0.0030 (8)
O31	0.0561 (9)	0.0458 (9)	0.0434 (9)	0.0058 (7)	0.0104 (8)	0.0035 (7)
O32	0.0540 (9)	0.0415 (8)	0.0426 (9)	0.0160 (7)	0.0147 (7)	0.0036 (7)
C33A	0.0264 (19)	0.0261 (13)	0.032 (2)	0.0037 (10)	0.0103 (15)	0.0011 (11)
C33B	0.033 (5)	0.026 (4)	0.030 (7)	0.006 (3)	0.003 (3)	0.001 (3)

### 1-(3,4-Dimethoxybenzyl)-6,7-dimethoxyisoquinoline–fumaric acid (2/3) (I) Geometric parameters (Å, º)

**Table:**

N1—H1	0.8800	C21—H21A	0.9800
N1—C2	1.328 (3)	C21—H21B	0.9800
N1—C10	1.350 (3)	C21—H21C	0.9800
C2—C3	1.415 (3)	O22—C23	1.432 (3)
C2—C11	1.496 (3)	C23—H23A	0.9800
C3—C4	1.419 (3)	C23—H23B	0.9800
C3—C8	1.416 (3)	C23—H23C	0.9800
C4—H4	0.9500	O24—C25	1.433 (3)
C4—C5	1.364 (3)	C25—H25A	0.9800
C5—C6	1.434 (3)	C25—H25B	0.9800
C5—O22	1.351 (2)	C25—H25C	0.9800
C6—C7	1.372 (3)	C34—O35	1.324 (2)
C6—O24	1.351 (2)	C34—O36	1.211 (3)
C7—H7	0.9500	C34—C37	1.480 (3)
C7—C8	1.416 (3)	O35—H35	0.8400
C8—C9	1.420 (3)	C37—C37^i^	1.331 (4)
C9—H9	0.9500	C37—H37	0.9500
C9—C10	1.361 (4)	C38—O39A	1.280 (4)
C10—H10	0.9500	C38—O39B	1.235 (5)
C11—H11A	0.9900	C38—O40A	1.231 (4)
C11—H11B	0.9900	C38—O40B	1.300 (5)
C11—C12	1.520 (3)	C38—C41A	1.476 (6)
C12—C13	1.398 (3)	C38—C41B	1.533 (10)
C12—C17	1.383 (3)	O39A—H39A	0.8400
C13—H13	0.9500	O40B—H40B	0.843 (5)
C13—C14	1.386 (3)	C41A—C41A^ii^	1.362 (9)
C14—C15	1.413 (3)	C41A—H41A	0.9500
C14—O18	1.364 (3)	C41B—C41B^ii^	1.218 (17)
C15—C16	1.378 (3)	C41B—H41B	0.9500
C15—O20	1.368 (2)	C30—O31	1.248 (3)
C16—H16	0.9500	C30—O32	1.246 (3)
C16—C17	1.399 (3)	C30—C33A	1.504 (3)
C17—H17	0.9500	C30—C33B	1.555 (10)
O18—C19	1.432 (2)	C33A—C33A^iii^	1.322 (8)
C19—H19A	0.9800	C33A—H33A	0.9500
C19—H19B	0.9800	C33B—C33B^iii^	1.32 (2)
C19—H19C	0.9800	C33B—H33B	0.9500
O20—C21	1.427 (2)		
			
C2—N1—H1	118.2	O18—C19—H19C	109.5
C2—N1—C10	123.56 (18)	H19A—C19—H19B	109.5
C10—N1—H1	118.2	H19A—C19—H19C	109.5
N1—C2—C3	118.9 (2)	H19B—C19—H19C	109.5
N1—C2—C11	117.53 (17)	C15—O20—C21	116.92 (17)
C3—C2—C11	123.60 (18)	O20—C21—H21A	109.5
C2—C3—C4	121.08 (19)	O20—C21—H21B	109.5
C2—C3—C8	119.45 (18)	O20—C21—H21C	109.5
C8—C3—C4	119.47 (16)	H21A—C21—H21B	109.5
C3—C4—H4	119.8	H21A—C21—H21C	109.5
C5—C4—C3	120.47 (18)	H21B—C21—H21C	109.5
C5—C4—H4	119.8	C5—O22—C23	116.43 (14)
C4—C5—C6	120.03 (17)	O22—C23—H23A	109.5
O22—C5—C4	125.26 (18)	O22—C23—H23B	109.5
O22—C5—C6	114.68 (15)	O22—C23—H23C	109.5
C7—C6—C5	120.41 (16)	H23A—C23—H23B	109.5
O24—C6—C5	113.56 (16)	H23A—C23—H23C	109.5
O24—C6—C7	126.02 (18)	H23B—C23—H23C	109.5
C6—C7—H7	119.9	C6—O24—C25	117.25 (16)
C6—C7—C8	120.11 (19)	O24—C25—H25A	109.5
C8—C7—H7	119.9	O24—C25—H25B	109.5
C3—C8—C9	117.70 (18)	O24—C25—H25C	109.5
C7—C8—C3	119.43 (17)	H25A—C25—H25B	109.5
C7—C8—C9	122.9 (2)	H25A—C25—H25C	109.5
C8—C9—H9	120.0	H25B—C25—H25C	109.5
C10—C9—C8	120.0 (2)	O35—C34—C37	113.63 (18)
C10—C9—H9	120.0	O36—C34—O35	124.4 (2)
N1—C10—C9	120.4 (2)	O36—C34—C37	121.98 (19)
N1—C10—H10	119.8	C34—O35—H35	109.5
C9—C10—H10	119.8	C34—C37—H37	117.7
C2—C11—H11A	109.0	C37^i^—C37—C34	124.6 (2)
C2—C11—H11B	109.0	C37^i^—C37—H37	117.7
C2—C11—C12	113.08 (16)	O39A—C38—C41A	116.0 (3)
H11A—C11—H11B	107.8	O39B—C38—O40B	128.8 (6)
C12—C11—H11A	109.0	O39B—C38—C41B	121.7 (6)
C12—C11—H11B	109.0	O40A—C38—O39A	125.4 (3)
C13—C12—C11	119.44 (18)	O40A—C38—C41A	118.6 (3)
C17—C12—C11	121.0 (2)	O40B—C38—C41B	108.9 (6)
C17—C12—C13	119.51 (19)	C38—O39A—H39A	109.5
C12—C13—H13	119.6	C38—O40B—H40B	102 (4)
C14—C13—C12	120.86 (18)	C38—C41A—H41A	118.9
C14—C13—H13	119.6	C41A^ii^—C41A—C38	122.3 (7)
C13—C14—C15	119.3 (2)	C41A^ii^—C41A—H41A	118.9
O18—C14—C13	125.05 (18)	C38—C41B—H41B	118.9
O18—C14—C15	115.65 (18)	C41B^ii^—C41B—C38	122.2 (15)
C16—C15—C14	119.54 (19)	C41B^ii^—C41B—H41B	118.9
O20—C15—C14	114.80 (19)	O31—C30—C33A	110.7 (2)
O20—C15—C16	125.64 (17)	O31—C30—C33B	145.1 (5)
C15—C16—H16	119.6	O32—C30—O31	122.17 (17)
C15—C16—C17	120.76 (18)	O32—C30—C33A	127.1 (2)
C17—C16—H16	119.6	O32—C30—C33B	92.6 (5)
C12—C17—C16	120.0 (2)	C30—C33A—H33A	119.1
C12—C17—H17	120.0	C33A^iii^—C33A—C30	121.9 (4)
C16—C17—H17	120.0	C33A^iii^—C33A—H33A	119.1
C14—O18—C19	117.23 (17)	C30—C33B—H33B	122.1
O18—C19—H19A	109.5	C33B^iii^—C33B—C30	115.7 (13)
O18—C19—H19B	109.5	C33B^iii^—C33B—H33B	122.1
			
N1—C2—C3—C4	−178.96 (16)	C11—C12—C13—C14	177.90 (16)
N1—C2—C3—C8	2.3 (3)	C11—C12—C17—C16	−178.71 (16)
N1—C2—C11—C12	103.3 (2)	C12—C13—C14—C15	0.6 (3)
C2—N1—C10—C9	−1.0 (3)	C12—C13—C14—O18	−178.84 (17)
C2—C3—C4—C5	−177.63 (17)	C13—C12—C17—C16	−0.9 (3)
C2—C3—C8—C7	175.96 (16)	C13—C14—C15—C16	−0.4 (3)
C2—C3—C8—C9	−2.8 (3)	C13—C14—C15—O20	−179.14 (16)
C2—C11—C12—C13	127.20 (19)	C13—C14—O18—C19	−1.7 (3)
C2—C11—C12—C17	−55.0 (2)	C14—C15—C16—C17	−0.5 (3)
C3—C2—C11—C12	−77.7 (2)	C14—C15—O20—C21	−179.53 (17)
C3—C4—C5—C6	1.4 (3)	C15—C14—O18—C19	178.86 (16)
C3—C4—C5—O22	179.76 (16)	C15—C16—C17—C12	1.1 (3)
C3—C8—C9—C10	1.5 (3)	C16—C15—O20—C21	1.8 (3)
C4—C3—C8—C7	−2.8 (3)	C17—C12—C13—C14	0.1 (3)
C4—C3—C8—C9	178.42 (16)	O18—C14—C15—C16	179.10 (16)
C4—C5—C6—C7	−2.2 (3)	O18—C14—C15—O20	0.3 (2)
C4—C5—C6—O24	177.01 (16)	O20—C15—C16—C17	178.15 (16)
C4—C5—O22—C23	−2.8 (3)	O22—C5—C6—C7	179.25 (16)
C5—C6—C7—C8	0.5 (3)	O22—C5—C6—O24	−1.5 (2)
C5—C6—O24—C25	−172.83 (16)	O24—C6—C7—C8	−178.63 (17)
C6—C5—O22—C23	175.66 (18)	O35—C34—C37—C37^i^	10.4 (3)
C6—C7—C8—C3	2.0 (3)	O36—C34—C37—C37^i^	−168.9 (2)
C6—C7—C8—C9	−179.28 (17)	O39A—C38—C41A—C41A^ii^	176.4 (4)
C7—C6—O24—C25	6.3 (3)	O39B—C38—C41B—C41B^ii^	−12.6 (10)
C7—C8—C9—C10	−177.25 (18)	O40A—C38—C41A—C41A^ii^	−5.6 (6)
C8—C3—C4—C5	1.1 (3)	O40B—C38—C41B—C41B^ii^	175.7 (8)
C8—C9—C10—N1	0.4 (3)	O31—C30—C33A—C33A^iii^	−171.1 (3)
C10—N1—C2—C3	−0.3 (3)	O31—C30—C33B—C33B^iii^	7.3 (12)
C10—N1—C2—C11	178.75 (18)	O32—C30—C33A—C33A^iii^	10.5 (4)
C11—C2—C3—C4	2.0 (3)	O32—C30—C33B—C33B^iii^	−176.6 (8)
C11—C2—C3—C8	−176.73 (16)		

Symmetry codes: (i) −*x*+1, −*y*+2, −*z*; (ii) −*x*+2, −*y*+2, −*z*+2; (iii) −*x*+2, −*y*+2, −*z*+1.

### 1-(3,4-Dimethoxybenzyl)-6,7-dimethoxyisoquinoline–fumaric acid (2/3) (I) Hydrogen-bond geometry (Å, º)

**Table:**

*D*—H···*A*	*D*—H	H···*A*	*D*···*A*	*D*—H···*A*
N1—H1···O31	0.88	2.20	3.034 (3)	159
N1—H1···O32	0.88	2.18	2.919 (2)	141
O35—H35···O31	0.84	1.83	2.617 (2)	156
O39*A*—H39*A*···O32	0.84	1.67	2.502 (4)	169

### Selected geometric parameters (Å) for fumaric acid *A*, *B*, and *C* in compound (I)

**Table:**

C30–O31	1.248 (2)
C30–O32	1.246 (3)
C34–O35	1.324 (3)
C34–O36	1.211 (2)
C38–O39A^a^	1.280 (5)
C38–O40A^a^	1.231 (6)
